# Application of fenitrothion on *Heteropneustes fossilis* causes alteration in morphology of erythrocytes via modifying hematological parameters

**DOI:** 10.1016/j.toxrep.2022.04.010

**Published:** 2022-04-16

**Authors:** Rifat Farjana Ritu, SM Majharul Islam, Harunur Rashid, Shahroz Mahean Haque, Ilham Zulfahmi, Kizar Ahmed Sumon

**Affiliations:** a Department of Fisheries Management, Faculty of Fisheries, Bangladesh Agricultural University, Mymensingh 2202, Bangladesh; b Department of Fisheries Resources Utilization, Faculty of Marine and Fisheries, Syiah Kuala University, Indonesia

**Keywords:** Organophosphate insecticide, LC_50_, Blood biomarkers, Aquaculture species, Erythrocytic abnormalities

## Abstract

In Bangladesh, the extensive use of fenitrothion on crops and in aquaculture ponds inevitably threatens a range of aquaculture species, including fish, owing to stress responses and physiological disturbances. The present study elucidated the potential toxic effects of fenitrothion on the blood biomarkers (haemato-biochemistry and structure of erythrocytes) of stinging catfish (*Heteropneustes fossilis*), a commercially significant aquaculture species. Fish were exposed to four sub-lethal concentrations (0%, 10%, 20%, and 40% of the 96-h LC_50_ value) of fenitrothion in triplicate and observed on the 7th, 14th, 21st, and 28th day following exposure. With increasing fenitrothion concentration, blood glucose and white blood cell levels increased significantly; in contrast, hemoglobin, red blood cell, and packed cell volume substantially decreased. However, the mean corpuscular volume and mean corpuscular hemoglobin did not change significantly during the exordial period (0–7 d); although, at a later stage, changes were observed. Frequencies of observed erythrocytic nuclear abnormalities, such as degeneration, bi-nucleus, micronucleus, notch nucleus, and nuclear bridge and erythrocytic cellular abnormalities, such as echinocytes, fusion, elongation, and tear drop morphology increased significantly in a concentration-dependent manner. Differences between the control individuals and those individuals under treatment were considered insignificant for twin cells on the 14th day of exposure. The study showed the pernicious impact of the effects of fenitrothion on *H. fossilis* through physiological alteration, which is likely to pose challenges for aquaculture production.

## Highlights

• Stinging catfish is highly prone to fenitrothion used in croplands and fish ponds.
• The 96-h LC_50_ of fenitrothion for stinging catfish was 8.3 mg/L.
• Blood glucose and white blood cell increased significantly.
• Hemoglobin, red blood cell and packed cell volume significantly decreased.
• Exposure to fenitrothion resulted in abnormalities of erythrocyte structure.

## Graphical Abstract

**Figure:**
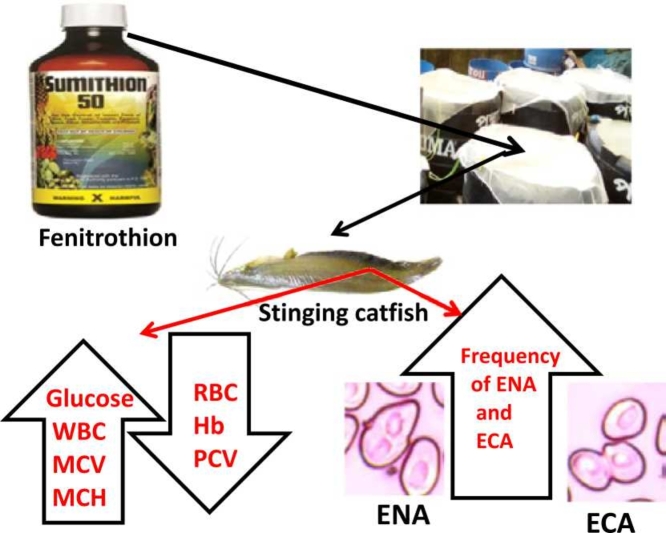

## 1 Introduction

Agricultural intensification is characterized by a greater usage of pesticides [1], and one-third of the global agricultural production is estimated to be protected from the effects of pests by their usage [2]. However, the serious consequences arising from the misuse, overuse, and error in application of 5 billion kg of pesticides globally every year [3] have been extensively debated and studied to understand the residual impact on the environment when exposed to these chemicals [4], [5] which affect the biodiversity, food safety, and ultimately human health as well as insecticide resistance [6], [7], [8]. Waterbodies that are close to agricultural lands and the communities residing on them are a major concern for direct and/or indirect exposure to these pesticides at lethal and sub-lethal concentrations when they exceed threshold levels [9]. Although pesticides comply with the gap for huge food demands, their more pronounced effects on non-target aquatic organisms, ranging from microalgae to fish [10], [11], [12], [13], cannot be ignored. In Bangladesh, a critical situation was highlighted when Akhter (2016) exposed that more than 90% of farmers were using pesticides on their crops, and approximately 47% were accused of overuse [3]. According to Miller [13], 98% of these pesticides come in contact with non-target organisms, including aquatic organisms.

Of principal importance in this study is fenitrothion, a type II synthetic organophosphate pesticide that has emerged as one of the replacements for organochlorines in Bangladesh. This pesticide is used extensively against locusts and grasshoppers in agricultural crop production [16], [17]. Public health programs against malaria and dengue have also addressed the extensive use of fenitrothion in Bangladesh [18]. From croplands, it makes its way into aquifers and aquatic environments through surface run-off, ground-water leaching, direct deposition, washing of application tools, and/or aerial spraying when used irresponsibly [1], [19], [20], [21], [22]. Moreover, in aquaculture settings, this pesticide is used extensively to control tiger bugs (*Cicindela* spp.) prior to stocking ponds with fish fry and juveniles [12]. Wherever used, it eventually makes its way into aquatic environments and has a negative impact on water quality [12], [23]. In water, fenitrothion affects the food and feeding habits of fish, benthos, and crustaceans and causes a reduction in the primary and secondary production of ponds in a direct and indirect manner [18]. The fenitrothion residue raises concerns regarding reproduction, structural development [24], [25], physiological mechanisms, and the activities and behavior [26] of fish, and thus, results in their survival rates being challenged.

Bangladesh, which is blessed with natural resources, prolific waterbodies, and a favorable climate, ranks 5th in global aquaculture production with an average growth rate of 5.26% per year for the last 10 years [27], [28], [29], [30], [31]. Along with shrimp farming and trading in coastal region of Bangladesh [13], [32], [33], [34], finfish farming in Bangladesh has expanded as a lucrative sector since the start of the 21st century [35], [36], [37], [38], [39]. Stinging catfish (*H. fossilis*), native to South Asian countries [40], has been proving its benefits in aquaculture production [27]. The simple breeding and shelter methods [41], nutritional and medicinal values [40], [42], and higher consumer preference [42], [43] of this catfish have resulted in its widespread adoption by farmers. However, most aquaculture ponds that are close to and/or influenced by agricultural lands are subjected to pesticide pollution in Bangladesh [18]. Stinging catfish, specifically its larval and juvenile stages, are more prone to *Cicindela* infestation. Farmers have found that fenitrothion and its derivative products are useful in curtailing production losses but it is often used in an irresponsible manner. Unfortunately, its most frequent use [17] in the monsoon season, as evidenced by the fact that its greatest concentration is found in water and sediments during the wet season [21], coincides with the breeding period of *H. fossilis*. Hence, the potential deadly impacts of fenitrothion on the juvenile stinging catfish have been hypothesized. Long-term exposure to this organophosphate pesticide reduces aquaculture production through behavioral changes in feeding habits [10], increased larval mortality [18], [44], changes in metabolism [45], and physiological dysfunction [46], [47]. Observed external deformities [18] may also contribute to the negative attitudes of consumers towards fenitrothion-exposed stinging catfish.

Blood directly linked to the branchial surface can be used to trace any deviation in water quality by reflecting it in the tissues, and thus, this is considered an essential biomarker. This excellent stress indicator is widely considered to reveal the potential toxic stress and health status of fish [44], [47], [48]. The erythrocytes of fish can readily respond to pollutants, and irregularity assessments of the cellular and nuclear morphology of erythrocytes has been used in pathophysiological studies [47], [49], [50], [51], [52]. The formation and development of micronuclei in erythrocytes is largely influenced by the toxicities arising from different contaminants, suggesting physiological stress syndromes in aquatic animals [53]. As a result, blood biochemical parameters and erythrocytic morphology are constantly used as potential analytical indicators for predicting fish health status in toxicity tests [47].

To date, an array of evidence on the adverse effects of fenitrothion on hemato-biochemical parameters and erythrocytic abnormality of eels [54], [55], common carp [46], striped catfish [44], and zebrafish [47] have been documented. Furthermore, [18] the effect of the toxicity of sumithion (a fenitrothion derived product) has been assessed on malformations of *H. fossilis* at the larval stage. However, the lack of information on fenitrothion pesticides has necessitated the assessment of blood biochemical parameters and patterns of cellular and nuclear abnormalities of erythrocytes to understand fenitrothion-induced toxicity in *H. fossilis*.

## 2 Materials and methods

### 2.1 Experimental fish holding and pesticide collection

Healthy adult stinging catfish of mixed sexes (n = 400; body weight = 30.6 ± 8.2 g; body length = 11.3 ± 3.7 cm) were collected from local aquaculture ponds in Mymensingh District. The fish were transferred to PVC tanks for 21 d. PVC tanks were set up in the Wet Laboratory of the Faculty of Fisheries, Bangladesh Agricultural University. A natural photo-regimen (14/10 h light/dark cycle) was maintained. Fish were fed commercial feed containing 30% crude protein twice a day at 5%/kg body weight. The handling, maintenance, and experimental procedures were approved by the Animal Care and Use Committee of the Bangladesh Agricultural University Research System, Bangladesh Agricultural University, Bangladesh (Ethical approval number BAURES/2018/648).

Fenitrothion (as sumithion with 50% active ingredient) was purchased in liquid form from a local pesticide dealer at Mymensingh City, and the expiry date and seal were properly checked before use.

### 2.2 Estimation of lethal concentration values of fenitrothion for stinging catfish

To determine the lethal concentrations (LC_10_, LC_50_, and LC_90_) of fenitrothion for adult stinging catfish, a 96 h static bioassay system was carried out according to the guidelines of the Organization for Economic Cooperation and Development [56]. Groups of ten fish were placed in each of the 18 PVC tanks containing 200 L dechlorinated tap water. The fish were acclimatized for seven days, and a commercial diet was maintained throughout this period. However, feeding was discontinued 24 h before the start of pesticide exposure. Five different concentrations of fenitrothion (4, 8, 16, 32, and 64 mg/L) as well as a control (0 mg/L, only dechlorinated tap water) were applied in triplicate. A stock solution of 1 L that was subsequently added to the respective tanks was prepared by dissolving the specified amount of fenitrothion in distilled water to achieve the desired concentration (500 g/L). Each tank was closely monitored twice daily to ascertain the dead fish for the specific time intervals of 24, 48, 72, and 96 h. Fish were considered dead if there was no visible movement, and if touching of the caudal peduncle resulted in no reaction. Dead individuals were immediately removed from the tanks and the lethal concentration values were estimated using probit analysis [57].

### 2.3 Experimental design for haemato-biochemistry and erythrocyte structure study

Ten female stinging catfish were transferred into each of the 12 PVC tanks containing 200 L of de-chlorinated tap water and acclimatized under laboratory conditions for 21 d prior to pesticide exposure. The average body length and weight of the experimental fish were 13.5 cm and 38.2 g, respectively. The fish were exposed to four sub-lethal concentrations of fenitrothion based on the 96-h LC_50_ value of 8.3 mg/L (see Results section for details). The details of the control and treatment groups were as follows: 0% of the 96-h LC_50_ (control, 0 mg/L), 10% of the 96-h LC_50_ (≈ 0.8 mg/L), 20% of the 96-h LC_50_ (≈ 1.6 mg/L), and 40% of the 96-h LC_50_ (≈ 3.3 mg/L). Each control and treatment group was set up with three replicates. To provide sufficient oxygen, an aeration system was installed in each tank. The experiment lasted for 28 d. Fish were fed a commercial diet twice daily at 5%/kg body weight. Approximately 90% of the water was changed weekly, and new fenitrothion concentrations were applied. Unutilized feed and feces were siphoned out using plastic pipes daily. Water quality variables were monitored weekly and the ranges of the following parameters were: temperature (26.0–28.5 °C), dissolved oxygen (6.9–7.4 mg/L), pH (7.9–9.8), and total alkalinity (176–204 mg/L).

### 2.4 Haemato-biochemistry

Three fish from each group were sampled on the 7th, 14th, 21st, and 28th day from the first fenitrothion exposure for the blood biomarker study. The fish were anesthetized with clove oil (5 mg/L) to avoid suffering during blood collection. A heparinized plastic syringe was used to collect blood from their caudal peduncles. Blood glucose and hemoglobin (Hb) levels were measured using a digital EasyMate® GHb, blood glucose/hemoglobin dual-function monitoring system (Model: ET- 232, Bioptik Technology Inc., Taiwan, 35057). Red blood cell (RBC) and white blood cell (WBC) counts were performed using a Neubauer hemocytometer under a light microscope following the standard protocol. In accordance with the method used by Jain (1993), the packed cell volume (PCV) (Eq. 1), mean corpuscular volume (MCV) (Eq. 2), and mean corpuscular hemoglobin (MCH) (Eq. 3) were calculated using the following formulas:

(1) PCV = % Hb × 3

(2) MCV = (PCV/RBC in millions) × 10

(3) MCH = (% Hb/RBC in millions) × 10

### 2.5 Erythrocytic abnormalities

Nuclear and cellular abnormalities in the erythrocytes of stinging catfish were studied following the standard protocol adopted by Islam et al. [44]. In brief, collected blood samples were smeared onto glass slides and air-dried for 10 min. The glass slides were fixed with methanol for 10 min, stained with 5% Giemsa, and rinsed with distilled water. They were air-dried overnight and mounted with DPX. Three slides were prepared from each fish, and 2000 cells with both cellular and nuclear membranes were scanned from each slide using an electronic microscope (MICROS Austria, MCX100) at 100x magnification. Erythrocytic nuclear abnormalities (ENA) and erythrocytic cellular abnormalities (ECA) were characterized according to the specifications described in [59] and [49].

### 2.6 Data analyses

The linear or monotonic association between *H. fossilis* mortality and the exposure concentration of fenitrothion was expressed by a linear model driven scatter plot. A linear model and scatter plot were obtained by performing an analysis in R (RStudio Team 2018). Assessment of the significant difference (p < 0.05) between the treatment groups for different blood biochemical parameters, blood cell count, and frequencies of ECA and ENA was done with the execution of one-way analysis of variance (one-way ANOVA). A further Bonferroni test was used to determine significant differences (p < 0.05). Because the maximum parameters were small, this test calibrated the significance between the treatment groups for better justification. Statistical analyses were performed using IBM SPSS Statistics for Windows, Version 23.0 (Armonk, NY, USA).

## 3 Results

### 3.1 Lethal concentration values of fenitrothion for H. fossilis

**Fig. 1:**
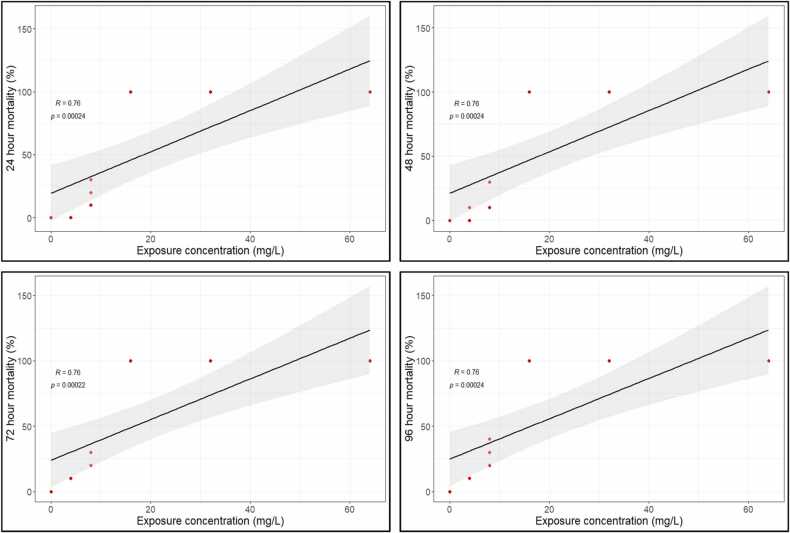
Regression graph showing relationship between exposure concentrations of fenitrothion and mortality of H. fossilis at different exposure periods.

Regression analysis between the mortality rate of stinging catfish and concentrations of fenitrothion showed a moderately strong relationship (R = 0.76) on all sampling days (Fig. 1). These lethal concentrations (LC_10_, LC_50_, and LC_90_) for different exposure periods (24, 48, 72, and 96 h) are shown in Table 1. The 96-h LC_50_ (with a 95% confidence interval) of fenitrothion for stinging catfish was determined to be 8.3 (7.2–9.7) mg/L.

**Table 1:** Lethal concentrations of fenitrothion (95% confidence interval) for H. fossilis for different time period.

Lethal concentration (mg/L)	Exposure time (h)			
	24	48	72	96
LC10	7.5 (5.5–8.2)	6.1 (3.7–7.5)	4.9 (3.7–5.9)	4.8 (3.6–5.8)
LC50	9.4 (8.5–14.3)	9.1 (7.4–11.7)	8.5 (7.3–9.9)	8.3 (7.2–9.7)
LC90	11.9 (10.0–33.6)	13.7 (10.9–24.8)	14.6 (12.0–20.0)	14.3 (11.8–19.6)

### 3.2 Blood biochemical parameters and blood cell count

**Fig. 2:**
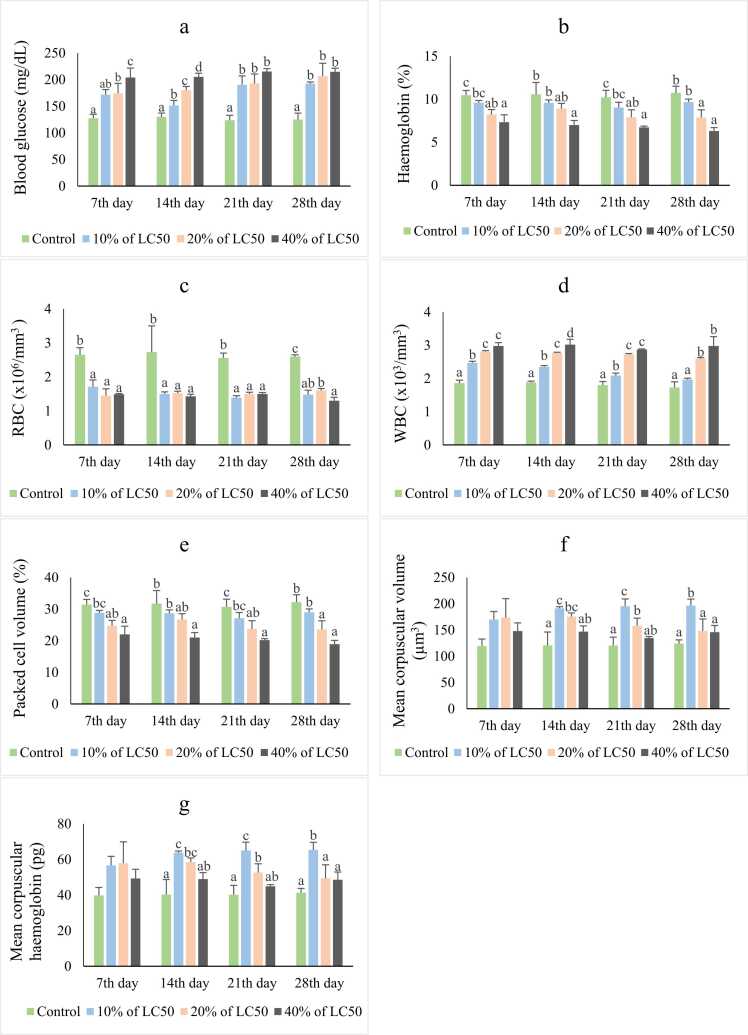
Changes in a) blood glucose level (mg/dL), b) haemoglobin (%), c) red blood cell count (x106/mm3), d) white blood cell count (x103/mm3), e) packed cell volume (%), f) mean corpuscular volume (µm3), g) mean corpuscular haemoglobin (pg) (mean ± SD) of H. fossilis after exposure to different concentrations of fenitrothion at different exposure periods are presented. Lowercase letters indicate statistically significant (p < 0.05) differences among the treatments.

The results showed a concentration-dependent increase in the aptitude for blood glucose and WBC, with a decreasing trend for hemoglobin, RBC, and PCV (Fig. 2). Cluttered conditions were observed for MCV and MCH. For blood glucose and WBC, exposure to 40% of LC_50_ of fenitrothion showed the highest peaks with a significant difference (p < 0.001) throughout the study period. Although significant augmentations (p < 0.001) between treatments for the first 14 d of exposure were visible, no intra-treatment stimulation for blood glucose was statistically significant (p > 0.05) on later sampling days. WBC observed in the 10% LC_50_ group at 28th day showed non-significant (p > 0.05) proliferation compared to the control. Hemoglobin and PCV significantly decreased (p < 0.001 and p < 0.05) throughout the exposure period resulting in the control and 40% LC50 group having the greatest and the lowest values, respectively. RBC showed non-significant reduction (p > 0.05) between treatments except on the 28th day, albeit all observations were significantly reduced (p < 0.05) compared to the control on that day. The highest MCH and MCV values were recorded in the group exposed to 10% LC50. While exposure to 20% of LC50 and 40% of LC50 of fenitrothion increased the values significantly (p < 0.05) on the 14th and 21st d, treatments were not significant (p = 0.057) compared to the control on the 7th day.

### 3.3 Erythrocytic nuclear abnormalities (ENA)

**Fig. 3:**
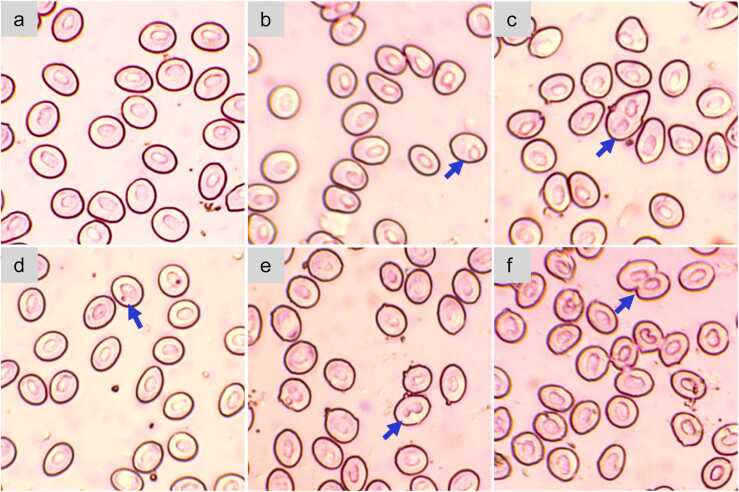
Blood smear of H. fossilis exposed to different levels of fenitrothion showing various types of erythrocytic nuclear abnormalities – a) normal nuclear morphology; b) nuclear degeneration; c) binucleated; d) micronucleus; e) notch nuclei; and f) nuclear bridge.

Several nuclear abnormalities in stinging catfish, including nuclear degeneration, bi-nuclei, micronucleus, notch nucleus, and nuclear bridges were revealed from blood samples from fish exposed to fenitrothion (Fig. 3). The frequencies of ENA followed an increasing trend with increasing fenitrothion concentrations. However, a significant increase (*p* < 0.05) was observed in the 20% LC50 group (except the micronucleus on the 7th day) and 40% LC_50_ group throughout the study period (Table 2). Fenitrothion exposure to 40% LC_50_ group posed significant effects (*p* < 0.001) on nuclear degeneration on the 21st day, on binucleated and micronuclei on the 21st day (*p* = 0.022 and p = 0.016) and 28th day (*p* = 0.001), on notch nuclei on the 7th day (*p* = 0.036), 21st day (*p* = 0.016), and 28th day (*p* = 0.011), and on the nuclear bridge at the end of the exposure period (*p* = 0.001) only. For all types of abnormalities, 40% LC_50_ group showed the highest frequency throughout the exposure period (Table 2).

**Table 2:** Frequencies of erythrocytic nuclear abnormalities (ENA) of H. fossilis exposed to different concentrations of fenitrothion for different sampling days.

Erythrocytic nuclear abnormalities (ENA)	Exposure time (days)	Treatments				
		Control	10% of LC50	20% of LC50	40% of LC50	*P* value
Nuclear degeneration	7	0.28 ± 0.04^a^	0.37 ± 0.04^a^	0.62 ± 0.06^b^	0.85 ± 0.11^c^	< 0.001
	14	0.24 ± 0.02^a^	0.37 ± 0.05^a^	0.65 ± 0.05^b^	0.81 ± 0.08^c^	< 0.001
	21	0.25 ± 0.05^a^	0.40 ± 0.05^b^	0.68 ± 0.08^c^	0.94 ± 0.04^d^	< 0.001
	28	0.29 ± 0.03^a^	0.45 ± 0.07^a^	0.70 ± 0.09^b^	1.08 ± 0.09^c^	< 0.001
Binucleated	7	0.31 ± 0.04^a^	0.33 ± 0.06^a^	0.53 ± 0.04^b^	0.68 ± 0.08^b^	< 0.001
	14	0.39 ± 0.03^a^	0.40 ± 0.07^a^	0.55 ± 0.10^ab^	0.71 ± 0.06^b^	0.001
	21	0.42 ± 0.07^a^	0.54 ± 0.05^ab^	0.61 ± 0.09^ab^	0.69 ± 0.11^b^	0.022
	28	0.36 ± 0.04^a^	0.57 ± 0.07^ab^	0.67 ± 0.12^bc^	0.88 ± 0.11^c^	0.001
Micronucleus	7	0.39 ± 0.06^a^	0.55 ± 0.02^a^	0.53 ± 0.04^a^	0.78 ± 0.10^b^	0.001
	14	0.40 ± 0.05^a^	0.44 ± 0.06^a^	0.59 ± 0.11^ab^	0.73 ± 0.12^b^	0.008
	21	0.44 ± 0.07^a^	0.55 ± 0.09^ab^	0.67 ± 0.13^ab^	0.82 ± 0.14^b^	0.016
	28	0.44 ± 0.06^a^	0.64 ± 0.08^ab^	0.84 ± 0.14^bc^	0.95 ± 0.10^c^	0.001
Notch nuclei	7	0.32 ± 0.08^a^	0.33 ± 0.06^ab^	0.39 ± 0.04^ab^	0.51 ± 0.10^b^	0.036
	14	0.30 ± 0.08^a^	0.43 ± 0.06^ab^	0.45 ± 0.08^ab^	0.57 ± 0.08^b^	0.016
	21	0.41 ± 0.03^a^	0.48 ± 0.06^a^	0.57 ± 0.07^ab^	0.67 ± 0.10^bc^	0.010
	28	0.52 ± 0.04^a^	0.72 ± 0.08^ab^	0.78 ± 0.09^b^	0.83 ± 0.12^b^	0.011
Nuclear bridge	7	0.34 ± 0.02^a^	0.40 ± 0.08^a^	0.64 ± 0.10^b^	0.98 ± 0.09^c^	< 0.001
	14	0.35 ± 0.05^a^	0.46 ± 0.10^a^	0.73 ± 0.08^b^	0.88 ± 0.08^b^	< 0.001
	21	0.46 ± 0.07^a^	0.57 ± 0.08^a^	0.81 ± 0.08^b^	0.95 ± 0.10^b^	< 0.001
	28	0.45 ± 0.05^a^	0.84 ± 0.13^b^	0.86 ± 0.10^b^	1.06 ± 0.12^b^	0.001

Results are presented as mean ± SD. Means in the same row indicated by different superscript letters are statistically significant (P < 0.05).

### 3.4 Erythrocytic cellular abnormalities (ECA)

**Fig. 4:**
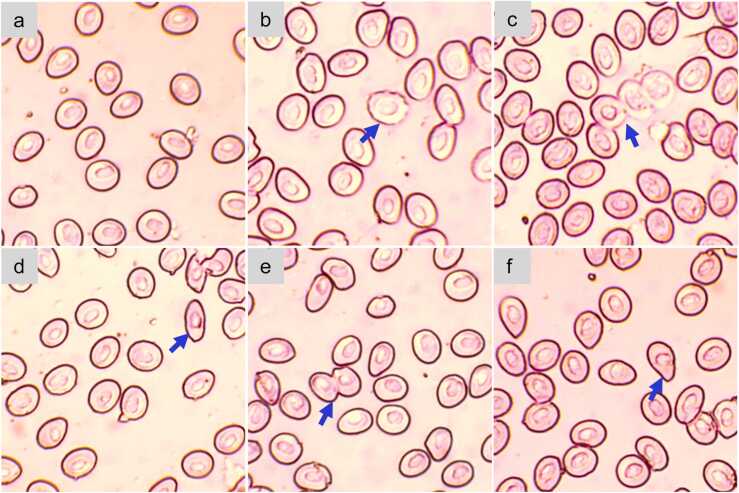
Blood smear of H. fossilis exposed to different levels of fenitrothion showing various types of erythrocytic cellular abnormalities – a) regular cell; b) echinocyte; c) fusion; d) elongated; e) twin; and f) tear drop.

Exposure to fenitrothion resulted in erythrocytic cellular abnormalities, including echinocytes, fusion, elongation, twinning, and tear drop morphology in the stinging catfish (Fig. 4). The observed frequencies of ECA increased with increasing concentrations of fenitrothion throughout the study period (Table 3). Up to 14 d of exposure, 10% of LC_50_ showed no significant effects (*p* > 0.05) for echinocytes, and 40% of LC_50_ caused the highest frequency of echinocytes. On later days, echinocytes in the 10% LC_50_ group increased significantly (p < 0.05), and 20% of LC_50_ had the highest echinocytes. While frequencies of fused cells were significant (*p* < 0.05) in all treatments compared to the control group, the highest frequencies were observed in the 20% LC_50_ group for the first 14 d and in the 40% LC_50_ group for the rest of the sampling days. Frequencies of cellular elongation, twin, and tear-dropped cells were significantly aggravated (*p* < 0.05) with the progression of fenitrothion concentrations for almost all sampling days, except for cellular elongation on the 21st day (p = 0.072) and for twin cells on the 14th day (p = 0.067).

**Table 3:** Frequencies of erythrocytic cellular abnormalities (ECA) of H. fossilis exposed to different concentrations of fenitrothion for different sampling days.

Erythrocytic cellular abnormalities (ECA)	Exposure time (days)	Treatments				
		Control	10% of LC50	20% of LC50	40% of LC50	*P* value
Echinocytic	7	0.21 ± 0.04^a^	0.27 ± 0.06^a^	0.36 ± 0.06^ab^	0.50 ± 0.14^b^	0.011
	14	0.29 ± 0.04^a^	0.40 ± 0.08^a^	0.44 ± 0.09^ab^	0.60 ± 0.06^b^	0.005
	21	0.40 ± 0.04^a^	0.51 ± 0.09^ab^	0.58 ± 0.10^ab^	0.67 ± 0.10^b^	0.024
	28	0.37 ± 0.14^a^	0.75 ± 0.11^b^	0.74 ± 0.09^b^	0.71 ± 0.17^ab^	0.023
Fusion	7	0.47 ± 0.05^a^	0.65 ± 0.08^ab^	0.71 ± 0.05^b^	0.69 ± 0.10^b^	0.017
	14	0.51 ± 0.13^a^	0.72 ± 0.07^ab^	0.81 ± 0.08^b^	0.75 ± 0.08^b^	0.019
	21	0.55 ± 0.13^a^	0.82 ± 0.10^ab^	0.86 ± 0.07^b^	0.87 ± 0.11^b^	0.018
	28	0.53 ± 0.07^a^	0.88 ± 0.15^b^	0.93 ± 0.13^b^	1.04 ± 0.15^b^	0.007
Elongated	7	0.49 ± 0.06^a^	0.51 ± 0.06^a^	0.59 ± 0.07^ab^	0.75 ± 0.10^b^	0.014
	14	0.48 ± 0.08^a^	0.57 ± 0.07^ab^	0.66 ± 0.08^ab^	0.71 ± 0.12^b^	0.041
	21	0.59 ± 0.05	0.67 ± 0.09	0.75 ± 0.14	0.83 ± 0.10	0.072
	28	0.55 ± 0.11^a^	0.78 ± 0.15^ab^	0.86 ± 0.14^ab^	0.95 ± 0.09^b^	0.022
Twin	7	0.42 ± 0.06^a^	0.48 ± 0.11^a^	0.57 ± 0.09^ab^	0.70 ± 0.05^b^	0.015
	14	0.53 ± 0.07	0.54 ± 0.12	0.66 ± 0.10	0.78 ± 0.13	0.067
	21	0.47 ± 0.06^a^	0.66 ± 0.10^ab^	0.76 ± 0.11^b^	0.81 ± 0.15^b^	0.022
	28	0.52 ± 0.08^a^	0.77 ± 0.12^ab^	0.91 ± 0.15^b^	0.93 ± 0.16^b^	0.019
Tear drop	7	0.40 ± 0.05^a^	0.50 ± 0.11^ab^	0.64 ± 0.11^ab^	0.69 ± 0.11^b^	0.025
	14	0.47 ± 0.13^a^	0.64 ± 0.07^ab^	0.79 ± 0.04^b^	0.73 ± 0.13^b^	0.018
	21	0.54 ± 0.08^a^	0.64 ± 0.09^ab^	0.80 ± 0.07^bc^	0.94 ± 0.14^c^	0.004
	28	0.53 ± 0.07^a^	0.77 ± 0.07^ab^	0.86 ± 0.14^b^	1.05 ± 0.17^b^	0.005

Results are presented as mean ± SD. Means in the same row indicated by different superscript letters are statistically significant (P < 0.05).

## 4 Discussion

In this doubly-faceted experiment, the first phase aimed to determine the lethal concentration values for stinging catfish studied; this was followed by a 28-day experiment that focused on hemato-biochemical parameters and erythrocytic morphology as potential biomarkers for assessing the toxicity of fenitrothion-exposed fish.

A wide range of pesticides are being used in Bangladesh, including, fenitrothion, malathion, acephate, chlorpyrifos, diazion, ethion, fenthion, quinalphos etc [21], [60]. A plethora of studies have reported mortality after treatment with fenitrothion and its derivative chemicals and determined the 96-h LC_50_ in different fish species. The mortality rate in our experiment increased in a concentration- and duration-dependent manner. The inability to survive at concentrations greater than 8 mg/L recorded here is lower than that determined by an earlier study with stinging catfish [61]. The 96-h LC_50_ value of fenitrothion (8.3 mg/L) determined in this study is far higher than those of the values determined for zebrafish larvae [12], [50] and striped catfish [44]; however, these values are similar for common carp [46]. Nevertheless, several earlier studies calculated much lower LC_50_ values for the effects of fenitrothion and its derivatives on *Oreochromis niloticus* [10], [62], [63], [64], *Salvelinus fontinalis,* and *Mugil cephalus* [65]. This supports the statement by Dutta et al. [54] on a higher LC_50_ for air-breathing fish than water-breathing fish, leading to the interpretation that fish with a larger water exchange surface area are affected more intensely [67], [68]. It was also reported that the gills were the most sensitive to and the primary route of pesticides entering fish. The early assumption that believes in the disruption of oxygen consumption in fish by fenitrothion [69] explains the lower sensitivity of stinging catfish to fenitrothion than fish with larger gill surfaces. However, the 96-h LC_50_ value of 11.8 mg/L for *H. fossilis* exposed to malathion observed by Durkin [58] remains lower than our current finding.

Blood glucose is considered the second most important biomarker after cortisol of pesticide-induced stress syndrome [71]. Significant elevation of blood glucose was observed in stinging catfish when exposed to fenitrothion. This agrees with the findings of previous studies on *Oreochromis niloticus* exposed to fenitrothion [63] and malathion [72], *Sarotherodon mossambicus* exposed to fenitrothion [73], *Heteropneustes fossilis* exposed to deltamethrin [74], and *Pangasianodon hypophthalmus* exposed to sumithion [44]. The augmentation of blood glucose levels indicates potential physiological stress in stinging catfish studied and might be attributed to the excessive transmission of glucose into the blood stream after the breakdown of glycogen through increased phosphorylase activity [75]. Fish might tend to satisfy extra energy demands through enhanced glucogenesis using the action of glucocorticoids and catecholamines. This causes hyperglycemia in fish [76], including *H. fossilis*, which is also linked to the disruption of carbohydrate metabolism [77].

Hemoglobin in fish exhibits pronounced roles in binding and transporting oxygen from the environment to internal organs in accordance with metabolic requirements [78]. A significant decrease in blood hemoglobin levels with increasing concentrations of fenitrothion indicates hypochromic microcytic anemia in *H. fossilis*. A similar trend was observed when striped catfish [44] and stinging catfish [61] were exposed to sumithion. In addition to facilitating hemoglobin, RBCs in fish play roles in gaseous exchange, immune responses, and modulation. This decrease in hemoglobin level and RBC count might be achieved due to disturbed homeostasis and impairment of hematopoietic organs, such as the kidney and spleen from where the erythrocytes of fish originate. The reduction in erythrocytes is in agreement with several observations on deltamethrin-treated *O. niloticus* [67], *P. hypophthalmus* [34], common carp [36], *Channa punctata* [68], and *H. fossilis* [49].

The WBC count in fish serves as a sensitive indicator of environmental and chemical stressors, and changes in number are considered as immuno-deviations. In the present study, fenitrothion augmented the WBC count in *H. fossilis*, suggesting momentous stress and tissue damage [82] and non-specific hampering of immunity [83] induced by fenitrothion toxicity. Significant increases in WBC counts observed in striped catfish [44] and common carp [83] exposed to sumithion and *Labeo rohita* exposed to triazophos [53] coincide well with our findings. Surprisingly, deltamethrin at subacute concentrations depressed the WBC count in *Oreochromis niloticus* [79]. Enhanced lymphopoiesis from lymph myeloid tissue might lead to an increased number of WBCs in toxic conditions. This observed stimulation interprets an attempt to produce antibodies at a higher level by fish as an immunological defensive mechanism to counter the existence and/or pathological impacts of fenitrothion [84].

While the MCV and MCH values followed a similar increasing trend, PCV significantly decreased with fenitrothion treatment in stinging catfish. These alterations in MCV, MCH, and PCV in our study correspond to the findings in European catfish exposed to diazinon [85]. Fenitrothion induced an increase in MCV and MCH which might be due to the acidic conditions resulting from exposure to it, causing RBCs to rupture [82]. An increase in MCH and MCV depicts higher circulation of larger RBCs and large numbers of swollen RBCs in blood, respectively [86]. In contrast, the lower PCV observed in the current study might have resulted from reduced Hb synthesis caused by fenitrothion in *H. fossilis*. Increased MCV and MCH and reduced PCV ultimately leaving evidence of macrocytic nesmochromic-type anemic conditions in fish [83].

Erythrocytes are highly sensitive to environmental or physiological deviations and can sustain evidence through imitation in cellular and nuclear morphology. Therefore, morphological anomalies of erythrocytes have been used to assess the extent to which a pollutant can affect the physiology of aquatic vertebrates. A blood smear of fenitrothion-treated fish revealed five types of ENA in a frequential hierarchy: nuclear bridge> nuclear degeneration> micronucleus> binucleated> notch nuclei. Frequencies and their order observed in the current study are slightly different from the observations with silver barb exposed to profenofos [51] and zebrafish exposed to fenitrothion [47]. Parallelism between chromosomal irregularities and the malformation of erythrocytic nuclei, as reported by Lim et al. [75], implies the potential genotoxic effects of fenitrothion. Toxicities arising from this potential genotoxicant can lead to DNA damage through alteration of the DNA base and cessation of DNA strands [76], which may later be translated into aneuploidy. Such evidence is visible in the case of *Channa punctata* treated with atrazine-based herbicides [88]. Notched cells found in the blood of stinging catfish studied might be formed by blistering of the nuclei due to failure of tubulin polymerization [52]. Increased frequencies of observed binucleated, nuclear bridge, and notch nuclei elucidate the mutagenic effects of fenitrothion responsible for the excessive production of caspase-activated DNAase, which causes cleavage of erythrocytic nuclei in fish [89]. Apart from the main nucleus, micronuclei appear as satellite nuclei in the cytosol of erythrocytes [89] as a consequence of unsuccessful anaphasing towards daughter nuclei formation with acentric chromosomes. The formation and severity of micronuclei in the current study are supported by several studies on striped catfish exposed to sumithion [44], zebrafish exposed to fenitrothion [47], silver barbs exposed to profenofos [51], and quinalphos [49]. Increased micronucleus frequency in erythrocytes signifies mitotic disturbance through chromosomal aberrations in *H. fossilis* induced by fenitrothion. Mis-pairing or non-pairing of DNA fragments could also contribute to increased micronucleus formation [90] upon fenitrothion exposure. Whatever the causes of origin, an increased number of micronuclei must increase the risk of degenerative diseases and genetic disorders through unstable chromosomes and disrupted DNA [47], [91].

In the present study, ECA in stinging catfish included echinocytes, fusion, elongated, twin, and tear drops exposed to fenitrothion. Khatun et al. [47] and Khan et al. [51] also documented similar ECA patterns in fenitrothion-exposed zebrafish and profenofos-exposed silver barbs, respectively. These deformations result from the modification of the plasma membrane of erythrocytes, rendering red blood cells more resistant to rupture while traversing through the micro vessels [50]. Fenitrothion may also contribute to atypical configurations by creating hypoxic conditions, leading to the corrosion of ATP. Increased malformation of erythrocytes might also be linked to higher lipid peroxidation in the blood of fish, as stated by Ghaffar et al. [43]. This enhanced peroxidation wrenches the lipid membrane; hence, the increase in permeability and flexibility of the membrane results in an increased number of echinocytes [51].

## 5 Conclusions

The 96-h LC_50_ of fenitrothion (8.3 mg/L) suggests moderate toxicity to *H. fossilis*. The results revealed that the sub-lethal concentration (10% of the 96-h LC_50_; 0.8 mg/L) of fenitrothion might cause alterations in haemato-biochemical parameters and erythrocytes of *H. fossilis* after 28 days of exposures. Cellular and nuclear anomalies revealed by the current study suggest that sub-lethal concentrations of fenitrothion are capable of causing genotoxic, mutagenic harm, and oxidative damage in fish. However, this study proposes future studies on chronic exposure to fenitrothion, with a particular focus on genotoxic and mutagenic actions in stinging catfish. Furthermore, we recommend an interdisciplinary intervention that encourages farmers to safely handle and use fenitrothion and its derivatives as a matter of urgency to avert fenitrothion-induced loss in *H. fossilis* production.

## Ethical considerations

The study was conducted in accordance with the ethical standard of the Animal Care and Use Committee of Bangladesh Agricultural University Research System (BAURES), Bangladesh Agricultural University, Bangladesh.

## CRediT authorship contribution statement

**Riffat Farzana**: Conceptualization, Methodology, Investigation, Formal analysis, Data curation, Writing – original draft. **SM Majharul Islam**: Investigation. **Harunur Rashid**: Conceptualization, Supervision, Fund acquisition, Project administration. **Shahroz Mahean Haque**: Conceptualization, Writing – review & editing; **Ilham Zulfahmi**: Conceptualization, Writing – review & editing; **Kizar Ahmed Sumon**: Conceptualization, Writing – review & editing, supervision, Funding acquisition, Resources.

## Declaration of Competing Interest

The authors declare that they have no known competing financial interests or personal relationships that could have appeared to influence the work reported in this paper.

## Data Availability

Data that supports the findings of the study are available from the corresponding author upon request.
